# Cyclin A2 Mutagenesis Analysis: A New Insight into CDK Activation and Cellular Localization Requirements

**DOI:** 10.1371/journal.pone.0022879

**Published:** 2011-07-28

**Authors:** Nawal Bendris, Bénédicte Lemmers, Jean-Marie Blanchard, Nikola Arsic

**Affiliations:** Institut de Génétique Moléculaire de Montpellier, Centre National de la Recherche Scientifique, Université Montpellier 2, Université Montpellier 1, Montpellier, France; Institut de Génomique Fonctionnelle de Lyon, France

## Abstract

Cyclin A2 is essential at two critical points in the somatic cell cycle: during S phase, when it activates CDK2, and during the G2 to M transition when it activates CDK1. Based on the crystal structure of Cyclin A2 in association with CDKs, we generated a panel of mutants to characterize the specific amino acids required for partner binding, CDK activation and subcellular localization. We find that CDK1, CDK2, p21, p27 and p107 have overlapping but distinct requirements for association with this protein. Our data highlight the crucial importance of the N-terminal α helix, in conjunction with the α3 helix within the cyclin box, in activating CDK. Several Cyclin A2 mutants selectively bind to either CDK1 or CDK2. We demonstrate that association of Cyclin A2 to proteins such as CDK2 that was previously suggested as crucial is not a prerequisite for its nuclear localization, and we propose that the whole protein structure is involved.

## Introduction

Cyclin-dependent kinases (CDK), that govern progression through the cell cycle, are mainly controlled by transient interactions with cyclin regulatory subunits and by reversible phosphorylation reactions [1], [2]. The specific functions of Cyclin A2 protein at different stages of the cell cycle are dependent upon its CDK partner. Cyclin A2 is essential for at least two critical points in the somatic cell cycle: during the S phase, when it activates CDK2, and during the G2 to M transition when it activates CDK1. Cyclin A2 localizes predominantly to the nucleus during the S phase where it regulates the initiation and progression of DNA synthesis [3]. Phosphorylation of components of the DNA replication machinery such as CDC6 by Cyclin A-CDK is believed to be important to ensure only one round of DNA replication per cell cycle. At the end of G2, Cyclin A2 relocalizes to the centrosomes in the cytoplasm, where it binds to the poles of mitotic spindles. A recent study demonstrated the implication of Cyclin A2 in the activation of the M-phase promoting complex composed of Cyclin B and CDK1 and that regulates G2-M transition [4]. Finally, phosphorylation of CDH1 by Cyclin A2-associated kinase prevents the formation of APC^CDH1^ and therefore delays cyclin B ubiquitination and degradation [5], [6]. Expression of Cyclin A2 is ubiquitous, and is essential for embryonic development, the null embryos dying at around day 5.5 *post-coitum* [7]. A recently published work using mice with conditional Cyclin A2 alleles, while pointing to the compensatory effect brought about by Cyclin E2 in Cyclin A2 deficiency, confirmed its absolute requirement not only in early embryonic development but also in the establishment of the hematopoietic lineage [8].

In multicellular organisms the activity of these cyclin–CDK complexes is precisely controlled at many levels, including cell-cycle regulated transcription, programmed proteolytic destruction, and even sub-cellular localization of cyclins. Cyclin A2 is tightly regulated at the transcriptional level during the cell cycle [9], [10]. Its mRNA level increases at the G1/S transition and peaks during the S phase. Cyclin A2 transcriptional regulation is modulated at the G1/S transition by peripheral cues such as growth factors, TGF-β as well as cell interactions with the extra-cellular matrix [11], [12]. Transcriptional repression of Cyclin A2 involves promoter-bound-complexes, which include E2F- and pRb-related proteins, as well as chromatin remodelling factors such as Brahma/SNF2α [10], [13].

The crystal structure of Cyclin A2, either isolated or associated to CDK2 [14], [15] is characterized by a rigid globular structure, except for its N-terminal part. The globular part of the protein is comprised of two sub domains: the cyclin box, conserved among cyclins, and the cyclin box fold. They both consist of 5 α helices. An additional α helix was found in the N-terminal half of the protein [14], [15]. Further studies based on mutagenesis approaches stressed the importance of the conserved cyclin box region, with a particular emphasis on the MRAIL motif localized within its hydrophobic patch [16] [17]. In addition, it was suggested that the N-terminal helix located upstream of this conserved region, interacts also with CDKs [18].

Immunofluorescence studies have shown that Cyclin A2 is a nuclear protein. However, it was also shown to play a role in the cytoplasm by regulating centrosome duplication [19]. In this context, it is worth mentioning the recently described protein, SCAPER that associates to Cyclin A2 exclusively in the cytoplasm. A full functional significance of this interaction remains unknown although it was suggested that SCAPER might buffer the cytoplasmic pool of Cyclin A2 [20]. These reports suggested both nuclear and cytoplasmic functions for Cyclin A2 and highlight the importance of its correct cellular localization. Cyclin A2 has no Nuclear Localization Signal (NLS) and it has been proposed that its nuclear localization correlates with its ability to form complexes with CDK partners [21]. In a recent study, Jackman *et al.* demonstrated that Cyclin A2 actually shuttles between the nucleus and cytoplasm thanks to its association with CDK2. Moreover, it was shown that nuclear import is predominant with respect to the export, resulting in a net nuclear localization as revealed by immunofluorescence analysis [17]. However, mechanisms controlling this translocation remain unknown due to the fact that CDK2 has no evident NLS signal either. It was also proposed that nuclear localization of Cyclin A2 correlates with its ability to interact with p107 [22]. In addition to p107, other Cyclin A2 partners with evident NLS such as p130, p21 or members of the E2F family were likely candidates for nuclear carriers [22].

In this study, using a mutagenesis approach on mouse Cyclin A2 cDNA, we delineated the domains and amino acids this protein required for its association to CDK1 and CDK2, for the induction of CDK kinase activity, and for its subcellular localization. We show that, aside from the hydrophobic region of the cyclin box (MRAIL), both the N-terminal and its α3 helices play also a crucial role in kinase activation. Interestingly, we were able to identify amino acid residues responsible for a differential binding of Cyclin A2 to CDK1 and CDK2. We also identified alleles of Cyclin A2 able to associate but not to activate these kinases, which is consistent with a two-step mechanism of CDK activation (binding-conformation change). We demonstrated that association to p21 or p107 is not a prerequisite for nuclear localization of Cyclin A2. Moreover, depletion of CDK2, p27 or both proteins had no effect on it either. Furthermore, truncation or deletion of various parts of Cyclin A2 failed to delineate a precise region mediating a proper nuclear localization, thus pointing to the involvement of the whole protein in this process.

## Results

### Construction of the different Cyclin A2 mutant cDNAs

**Figure 1:**
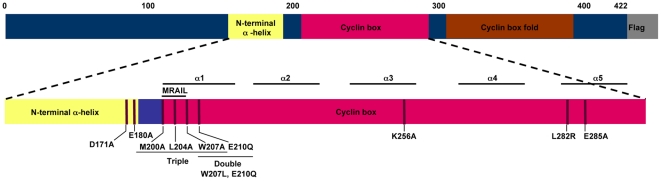
Schematic representation of mouse Cyclin A2 cDNA with the modifications introduced in this study. All constructs were C-terminally fused to 3 Flag tags.

In order to study critical amino residues necessary for Cyclin A2 interaction with its known partners, an intensive directed mutagenesis was performed. In addition to single amino acid substitutions, combinations of double or triple point mutations were performed. Mutations spanned the cyclin box (α1, α3 and α5 helices) as well as the N-terminal α helix (Figure 1). Mutations D171A and E180A (E167A and E176A in Xenopus Cyclin A respectively), as well as Triple, M200A, L204A, W207A (M210A, L214A, W217A in human Cyclin A2) were already described in the literature [16], [18]. All Cyclin A2 cDNAs were Flag-tagged.

### Partners binding and kinase-activating abilities of Cyclin A2 mutants

Capability of mutated Cyclin A2 protein to associate with its endogenous partners was tested in co-immunoprecipitation assays after transfection in NIH3T3 cells. Flag-tag was used to specifically precipitate mutant Cyclin A2 and discriminate it from the endogenous Cyclin A2 protein. For kinase activating assays, H1 histone was used as a template.

**Figure 2:**
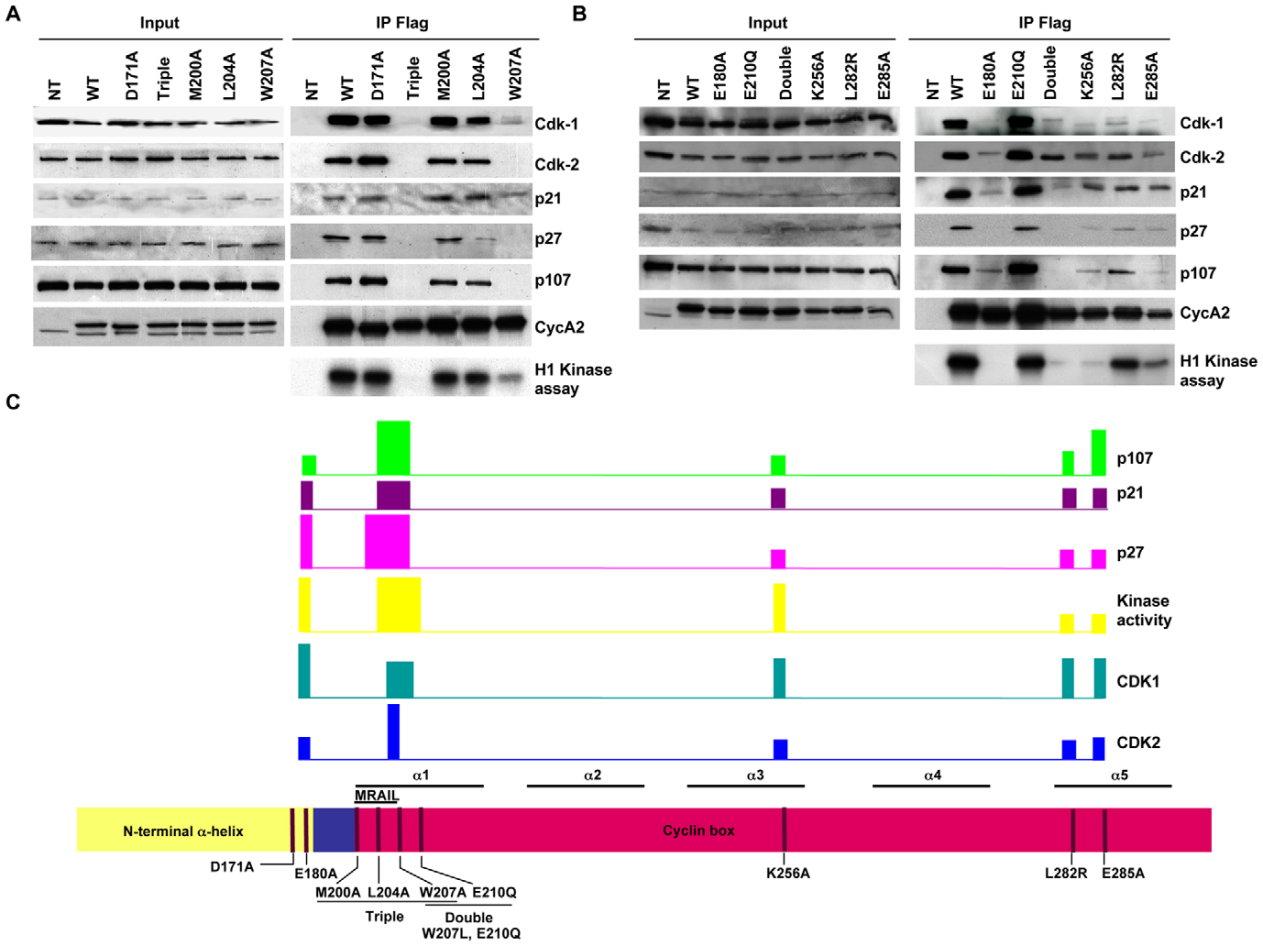
Analysis of the binding and kinase-activating properties of different Cyclin A2 alleles in NIH3T3 cells. WT, D171A, Triple (M200A, L204A and W207A), M200A, L204A, W207A (**A**), and WT, E180A, E210Q, Double (W207L, E210Q), K256A, L282R and E285A Cyclin A2 alleles (**B**). Immunoprecipitation using anti-Flag-agarose affinity gel was performed on NIH3T3 cells extracts after transfection of plasmids encoding the above-mentioned mutants. Immune complexes were either submitted to Western blotting to identify binding partners or analyzed for their histone H1-kinase activity. Schematic representation of the importance of the different Cyclin A2 domains for partners association and kinase-induced activity. The bars represent (high-medium-low according to their size) the importance of the indicated region of Cyclin A2 for its association with partners or kinase inducing activity (**C**).

Interestingly two kinases that associate with Cyclin A2, CDK2 and CDK1 demonstrated diverse amino-acids requirements for their binding. Crucial Cyclin A2 regions for these interactions were localized in the N-terminal α-helix, the hydrophobic region (MRAIL) and the α3 helix of the cyclin box with different importance for each kinase. First, E180A substitution abolished Cyclin A2 association with CDK1 while retaining to some extent its ability to bind to CDK2. However, this association was not sufficient to induce any kinase activity (Figure 2B). Interestingly, the corresponding mutation in *Xenopus* Cyclin A (E176E) abolished completely its binding to CDKs [18]. An additional difference with respect to the already published data was observed using D171A substitution (E167A in *Xenopus* Cyclin A). This mutation, proposed to discriminate between CDK1 and CDK2 association in *Xenopus levis* cells [18], was not effective in mammalian cells. Cyclin A2 mutation D171A had no effect in mouse cells, neither on its association with selected partners nor on its ability to activate CDKs (Figure 2A).

Furthermore, we investigated the effects of mutations in the MRAIL motif within the hydrophobic region of the cyclin box. Previous studies suggested different roles for this motif in Cyclin A2 functions. While Schulman and co-workers pointed to its importance in the association with RXL-containing proteins without affecting binding to CDKs, Jackman and colleagues suggested that mutations in the MRAIL domain also strongly affect the association to CDKs [16], [17]. To clarify the role of this domain, we created corresponding mutations in mouse Cyclin A2 in order to address their outcome on its properties. Actually, the triple substitution M200A, L204A, W207A, completely abolished any association with all Cyclin A2 partners tested in NIH3T3 cells and, as a consequence, impaired its kinase activating ability (Figure 2A). Our results with the Triple mutant are in agreement with a study performed by Schulman and co-workers with regards only to Cyclin A2 binding to proteins bearing an RXL motif (p21, p27 and p107) but not to that of CDK2 [16]. In the latter case, we confirmed that mutations in MRAIL also strongly affect associations to CDKs as suggested by Jackman and co-workers. Furthermore, we extended our analysis by evaluating the role of each single substitution within the compound mutation. Interestingly any single substitution of these three amino acids did not recapitulate the behavior of the Triple mutation with respect to partners binding and CDK-inducing ability (Figure 2 and Table 1). However, substitution W207A was discriminative for the binding of Cyclin A2 to CDKs as, under this condition, Cyclin A2 was still able to bind to CDK1 but not CDK2, and led to histone H1 phosphorylation (Figure 2A).

**Table 1:** Summary of binding affinities, kinase activating abilities and cellular localizations of Cyclin A2 alleles in mouse NIH3T3 cells.

	CDK 1(%)	CDK2(%)	p21(%)	p27(%)	p107(%)	Kinase activity(%)	Cellular localisation
WT	100	100	100	100	100	100	nuclear
D171A	100	100	100	100	100	88,8	NA
M200A	72,8	86,4	100	69,2	79,2	79,6	NA
L204A	57,2	72,4	100	15,2	65,6	71,9	predominant nuclear
W207A	15,3	1,2	79,2	2,3	0	16,7	predominant cytoplasmic
Triple (M200A,L204A, W207A)	0,4	0	0	0	0	0,9	predominant cytoplasmic
E180A	0	17,7	11,7	0	19,6	0	predominant cytoplasmic
E210Q	45,3	99,6	60,2	95,7	70	47,8	predominant nuclear
Double (W207L, E210Q)	9,2	66,4	8,2	0,9	1,8	0,51	predominant cytoplasmic
K256A	3,8	68,2	41,9	19,3	17	2,1	predominant nuclear
L282R	19,8	81,5	65,7	44,5	58,6	18,8	predominant nuclear
E285A	4,7	41,7	78,2	36,7	8,6	3,4	nuclear

Data are represented as percentage relative to WT control taken as 100% upon normalization to Cyclin A2 immunoprecipitation efficiency using densitometric analysis.

The most interesting observation obtained by mutagenesis analysis was the identification of amino acid residues in Cyclin A2 protein contributing to a differential association to CDKs. These residues were identified in the N-terminal helix (E180A) but also MRAIL domain (W207A). However, the most striking differences in discriminating amino acids essential for Cyclin A2 association to CDK1 and CDK2 were identified after performing mutations in the regions downstream of the MRAIL motif. The substitutions tested (Double mutation W207L, E210Q and single mutations K256A, L282R and E285A) had a moderate effect on Cyclin A2 ability to interact with CDK2. On the other hand, these mutations strongly affected CDK1 association to Cyclin A2. Thus, we showed that CDK1 and CDK2 interact differently with specific Cyclin A2 amino acid residues. Interestingly, even if the Double (W207L, E210Q) and single K256A mutants were still able to interact with CDK2 and weakly with CDK1 (K256A), their kinase-inducing activities were strongly affected (0,51% for the Double mutant and 2.1% for K256A as referred to 100% for WT Cyclin A2). All together these observations highlighted the importance of several domains for promoting kinase activity: N-terminal α-helix, MRAIL with adjacent domains, and cyclin box α3 helix.

Other Cyclin A2 partners were tested in this study and demonstrated diverse binding properties toward the different Cyclin A2 mutants. Mutations in the MRAIL domain (Triple, Double and W207A) severely impaired the binding to Cyclin A2 of proteins containing a RXL domain such as p27, p107 and p21. This is in accordance with previous results published by Schulman *et al.* where the corresponding human Cyclin A2 mutant (M210A, L214A, W217A) was shown to be defective in interacting with the above-mentioned partners. Surprisingly, mutations located in the N-terminal α-helix of Cyclin A2 (E180A) also strongly impaired association of p107 and p21 with Cyclin A2. We also were unable to detect any p27 association with this mutant under the same conditions. Finally, the amino acid substitutions located in α3 and α5 helices (K256A, L282R and E285A) reduced the association of the tested RXL proteins to Cyclin A2 without abolishing it. All together these data suggested that each Cyclin A2 partner tested in this study has particular requirements for its association with this protein. A schematic representation of the importance of the different Cyclin A2 domains for partners association and CDK activation is shown in figure 2C.

**Figure 3:**
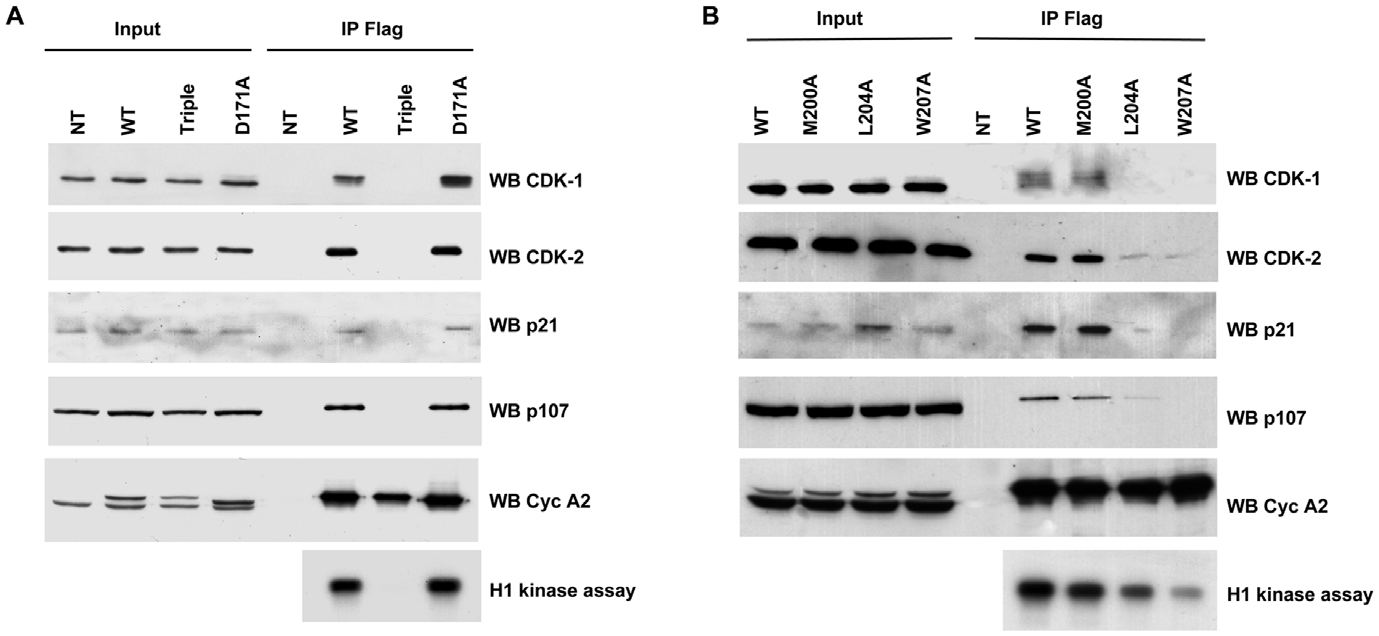
Analysis of binding and kinase-activating properties of different Cyclin A2 alleles in HEK 293 cells. WT, Triple (M200A, L204A, W207A), D171A (**A**) and M200A, L204A and W207A Cyclin A2 alleles (**B**). Immunoprecipitation using anti-Flag-agarose affinity gel was performed on HEK 293 cells extracts after transfection of vectors encoding the above-mentioned mutants. Immune complexes were either submitted to Western blotting to identify binding partners or analyzed for their histone H1-kinase activity.

In order to study the conservative relevance of these observations, mouse Cyclin A2 alleles were transfected into human HEK 293 cells and their association and kinase-activation ability were assessed. WT, Triple, M200A or D171A alleles harbored the same properties as in NIH3T3 cells. However, L204A and W207A alleles showed a different pattern in human with respect to mouse cells (Figure 3 and Table 2). Whereas the W207A allele associated with CDK1 but not with CDK2 in homologous cells, it completely switched its selectivity in human cells (binding to CDK2 but not CDK1). In addition, L204A allele demonstrated a reduced affinity to all partners tested in human cells compared to mouse cells, with a complete loss of affinity to CDK1.

**Table 2:** Summary of binding properties for some Cyclin A2 alleles in human HEK 293 cells.

	CDK 1(%)	CDK2(%)	p21(%)	p107(%)
WT	100	100	100	100
D171A	100	96	94,4	95
M200A	100	100	89,5	57,4
L204A	0	12,6	4,1	5,3
W207A	0	5	0,5	0
Triple (M200A,L204A, W207A)	0	0	0	0

Data are represented as percentage relative to WT control taken as 100% upon normalization to Cyclin A2 immunoprecipitation efficiency using densitometric analysis.

### Requirements for nuclear localization of Cyclin A2

**Figure 4:**
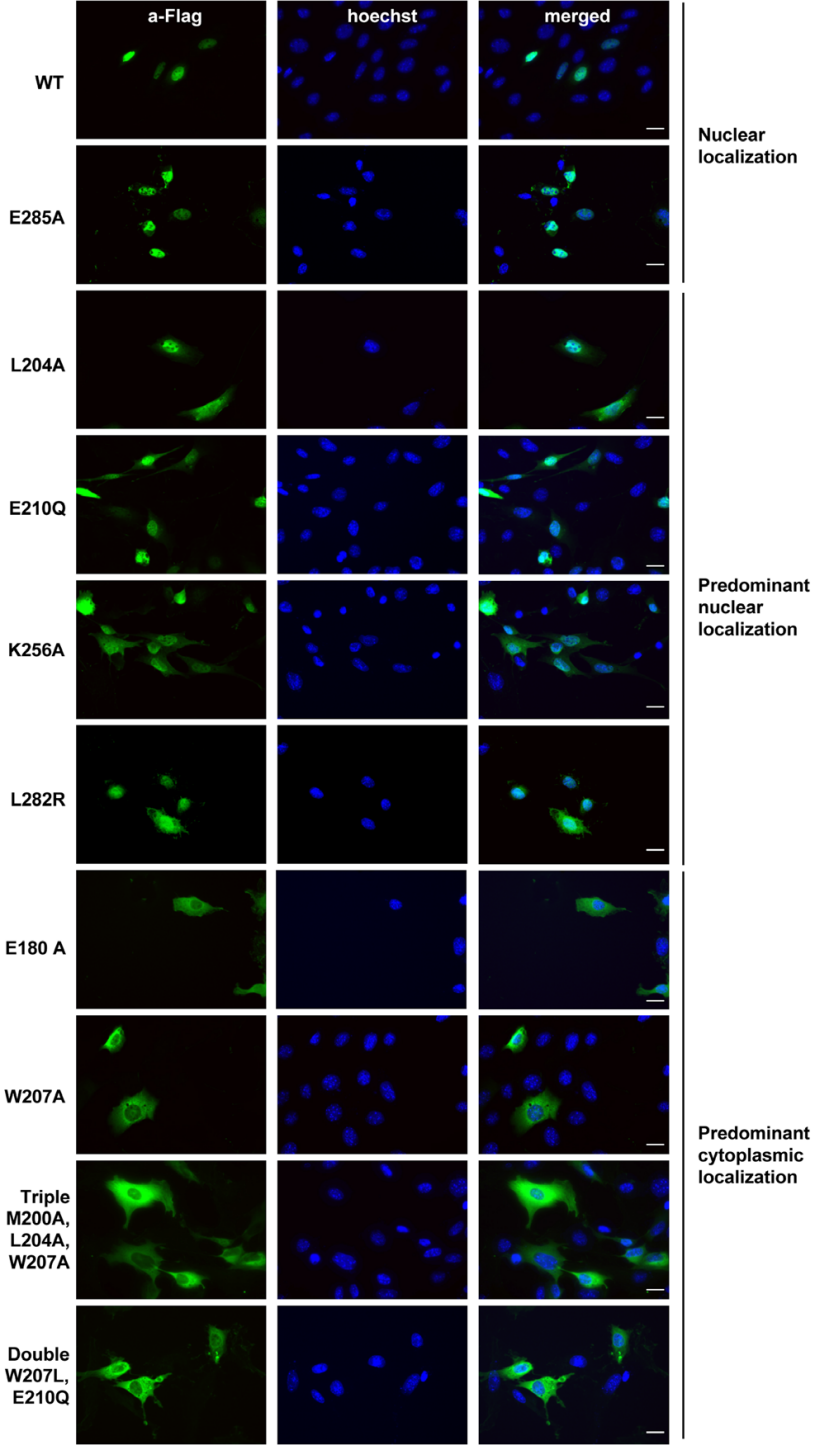
Immunofluorescence analysis of cellular localization for different Cyclin A2 alleles in NIH3T3 cells. WT, L204A, W207A, Triple (M200A, L204A, W207A), E180A, E210Q, Double (W207L, E210Q), K256A, L282R and E285A Cyclin A2 alleles. Staining performed using anti-Flag antibody (green) and Hoechst (blue) in NIH3T3 cells transfected with vectors encoding these Cyclin A2 alleles. Scale bar: 20 µm.

The cellular localization of Cyclin A2 mutants was determined by immunocytochemical analysis using an antibody against the Flag-tag as shown in Figure 4. This analysis showed different localizations for Cyclin A2 alleles. Namely, we were able to detect them either exclusively in the nucleus or in the cytoplasm, with an additional classes that exhibited a mixed but predominant nuclear or cytoplasmic localization. A summary of the sub cellular localization of all Cyclin A2 mutants is represented in Table 1. WT and E285A alleles demonstrated a clear exclusive nuclear localization, whereas L204A, E210Q, K256A and L282R were predominantly nuclear. On the contrary E180A, W207A, Triple (M200A, L204A, W207A) and Double (W207L, E210Q) alleles were located predominantly in the cytoplasm of the cells. Association to CDK2 has been suggested to be a prerequisite for nuclear localization of Cyclin A2. However, E180A and Double (W207L, E210Q) mutant alleles that associate with CDK2 were found predominantly cytoplasmic. The same was true for CDK1, since W207A and Double (W207L, E210Q) mutants while still able to bind this kinase, were predominantly cytoplasmic. Furthermore, p107 was suggested also to be a potential carrier of Cyclin A2 to the nucleus. Interestingly, E180A Cyclin A2 mutant, which is still able to bind p107 was found cytoplasmic. Similarly, p21, another Cyclin A2 partner with an evident NLS, was also suggested as its nuclear carrier [22]. Surprisingly, W207A, E180A and Double (W207L, E210Q) mutants, which are still interacting with p21, harbored a predominant cytoplasmic localization. Interestingly, only mutant alleles that maintain an affinity for both p27 and CDK2 (L204A, E210Q, K256A, L282R, E285A) were found with an exclusive or predominant nuclear localization.

**Figure 5:**
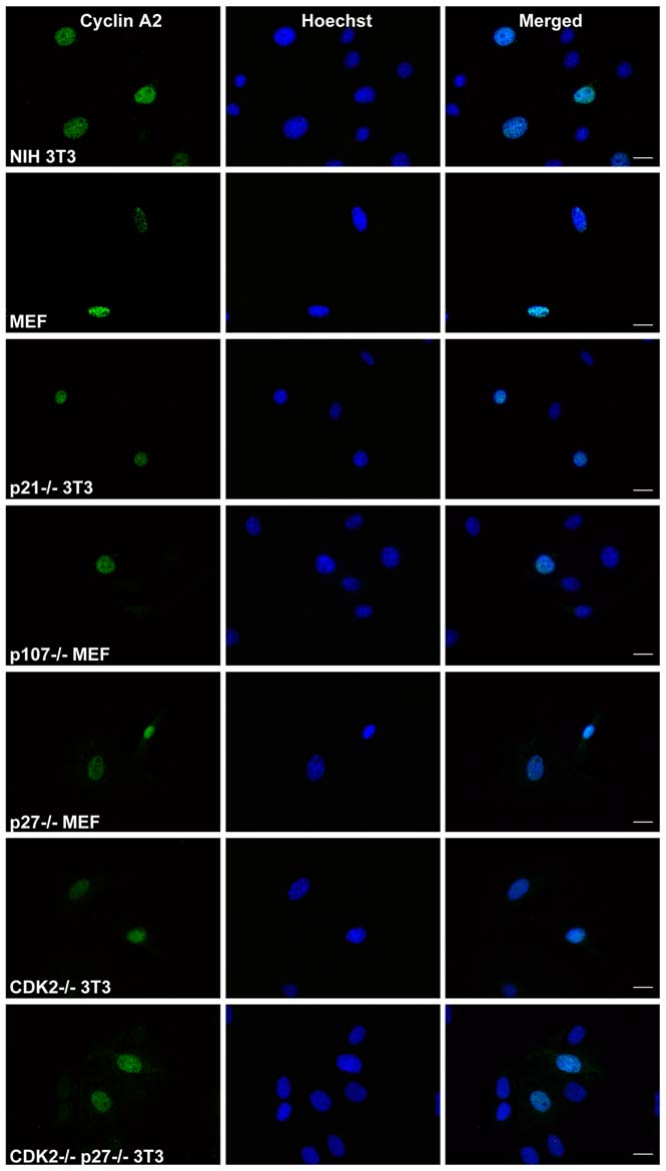
Immunofluorescent analysis of Cyclin A2 localization in *p21* ^−/−^, *p107* ^−/−^, *p27* ^−/−^, *CDK2* ^−/−^ and double *p27* ^−/−^ *CDK2* ^−/−^ genetic backgrounds. Staining was performed using anti-Cyclin A2 antibody (green) for endogenous Cyclin A2. Hoechst (blue) was used for nuclear staining. Scale bar: 20 µm.

In order to address the involvement of each Cyclin A2 binding partners for its nuclear localization, we resorted to the use of *p21* ^−/−^, *p27* ^−/−^, *p107* ^−/−^, *CDK2* ^−/−^ and double *p27* ^−/−^, *CDK2* ^−/−^ fibroblasts. We observed that cells devoid of p21 and p107 exhibit a nuclear localization of Cyclin A2 (Figure 5). Surprisingly, neither the depletion of p27 nor that of CDK2 had any effect on Cyclin A2 nuclear localization, although the latter was suggested as a main carrier for nuclear import. More surprisingly, depletion of both p27 and CDK2 did not result in a dramatic alteration in Cyclin A2 localization (Figure 5) even though a small proportion of it was consistently found cytoplasmic (Figure 5). Identical results were obtained in tranfection experiments using Flag tagged WT Cyclin A2 allele followed by immunofluorescence analysis with an anti-Flag antibody (data not shown).

**Figure 6:**
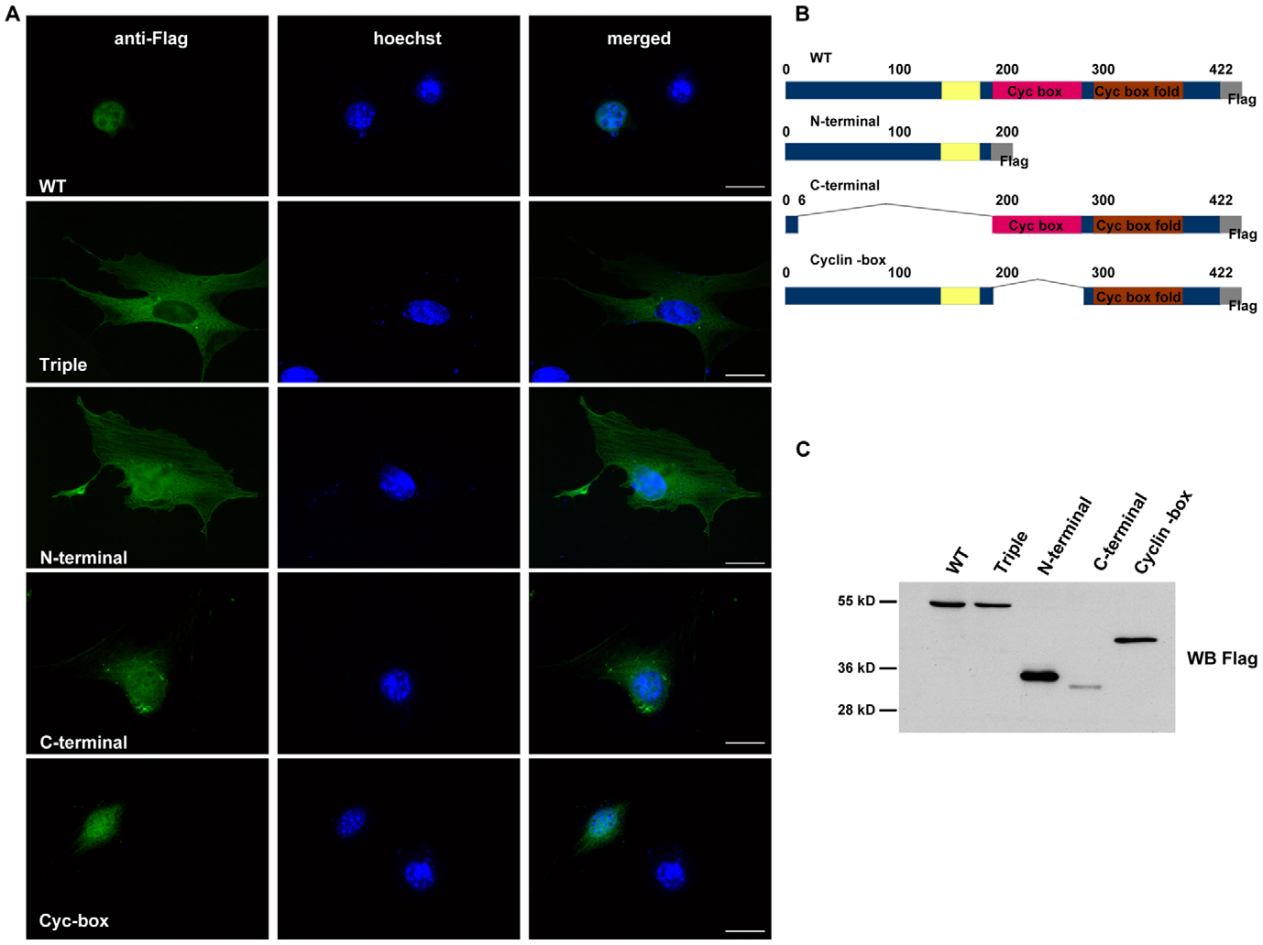
Immunofluorescence analysis of localization for Cyclin A2 deletion mutants. Immunofluorescent staining for Cyclin A2 deletion mutants localization using anti-Flag antibody (**A**). Schematic representation of Cyclin A2 deletion mutants (**B**). Western blot analysis of Cyclin A2 deletion mutants expression using anti-Flag antibody (**C**). Scale bar: 20 µm.

Interestingly, a deletion analysis failed to unveil any specific region of Cyclin A2 responsible for its nuclear localization. Both N- and C-terminal deletion mutants demonstrated both nuclear and cytoplasmic localization, although some differences were observed. Namely, the N-terminal domain was predominantly cytoplasmic whereas the C-terminal counterpart was found predominantly nuclear (Figure 6). These data suggested that although the C-terminal moiety of the protein was of crucial importance for its proper nuclear localization, additional domains in the N-terminal part participated to it.

## Discussion

Previous structural studies highlighted the importance of the hydrophobic region (MRAIL motif) of the first α helix within the cyclin box for molecular contacts of Cyclin A2 with CDK2 [14], [15]. Furthermore, mutagenesis carried out on the human Cyclin A2 pointed to amino acid residues M210A, L214A, W217A, as crucial for its association with RXL-containing partners without affecting its CDK2 kinase-activating ability [16]. However, subsequent studies identified the MRAIL hydrophobic patch of Cyclin A2 as important for its association with CDKs [17]. We constructed the corresponding mouse Cyclin A2 mutant (M200A, L204A, W207A corresponding to M210A, L214A, W217A human Cyclin A2) and addressed its association to some Cyclin A2 partners, its kinase activating properties and subcellular localization. This allele lost its ability to associate with any tested Cyclin A2 partner and to induce any kinase activity. This is in agreement with the studies of Schulman *et al.* and Jackman *et al.* that reported a defective interaction of the corresponding human Cyclin A2 mutant with RXL containing proteins (p21, p27 and p107) and CDKs, respectively. In addition, we confirmed the observation of Jackman and colleagues on the cytoplasmic localization of this Cyclin A2 mutant. Interestingly, our analysis demonstrated that the single substitutions (M200A, L204A or W207A) did not recapitulate the behavior of the Triple mutation with regards to all properties examined so far. An identical observation was obtained for the Double W207L, E210Q substitution. Interestingly, some amino acid residues contributed differently to the association of Cyclin A2 to CDKs. Thus, the W207A substitution heavily affected the interaction of Cyclin A2 with CDK2 but in less extent CDK1.

Previous work also stressed the importance of the N-terminal α helix located upstream of the cyclin box for Cyclin A2 binding properties in *Xenopus levis* [18]. We have created the corresponding mutations (D171A and E180A in mouse by comparison to E167A and E176A in *Xenopus levis*). The E167A substitution was described as discriminative for CDK1 and CDK2 binding in *Xenopus*. However, the corresponding D171A mutation, as shown in this study, had no effect on Cyclin A2 binding to CDK1 or CDK2 in mammalian cells. On the other hand, we show that the E180A mutant was deficient in binding CDK1 while still able to associate to CDK2 even with a reduced efficiency. Although being still able to bind to CDK2, this allele was not inducing any kinase activity. To our knowledge, this is the first demonstration of the functional importance of the N-terminal α helix for Cyclin A2 induced kinase activity in mammalian cells.

Substitutions in the central and C-terminal parts of the cyclin box (α helices 3 and 5 respectively) unveiled the importance of these regions for Cyclin A2 function. Each of these mutations significantly reduced, but not totally eliminated, the binding of Cyclin A2 to its partners, resulting in a low kinase-activation potential. However, cellular localization was not affected. Moreover, these mutations strongly affected the association to CDK1, which was not the case for CDK2, suggesting different requirements for the association with these two kinases.

This study provides evidences that, although sharing some common features for binding to Cyclin A2, CDK1 and CDK2 have particular requirements for this association. Actually, we describe a substitution able to discriminate between CDK1 and CDK2. Namely, the W207A allele binds only to CDK1, whereas the E180A mutant targets CDK2. This is also confirmed by substitutions within the α3 and α5 helices from the cyclin box as discussed above. We also demonstrated that binding to CDKs does not automatically lead to their activation. Alleles E180A, Double (W207L, E210Q) and K256A were able to bind to some extent one or both CDKs without a significant induction in their kinase activity.

This study sheds a new light on Cyclin A2 cellular localization. Previous publications suggested a role for CDK2 in this process although the mechanism remained elusive due to the fact that this kinase, just like Cyclin A2, has no evident nuclear localization signal. It was also proposed that Cyclin A2 partners with genuine NLS signal, such as p107, p130, p21 or members of E2F transcription factor family could contribute to its import into the nucleus. Here we demonstrate that binding of Cyclin A2 to p21 or p107 is not a prerequisite to its nuclear localization. Actually, cells devoid of these proteins show a clear nuclear localization of Cyclin A2. However, our experiments couldn't completely rule out CDK2 and p27 participation since in their absence some small amount of Cyclin A2 was detected in the cytoplasm. Furthermore, we investigated the roles of individual Cyclin A2 domains in its nuclear localization. Since both N- and C-terminal deletions were found in the nucleus as well as in the cytoplasm, even though the C-terminal part appeared to have a predominant role in nuclear localization, we propose that the whole protein moiety is involved in its proper subcellular localization.

## Materials and Methods

### Plasmids and cell lines

Mouse Cyclin A2 cDNA was obtained from NIH3T3 cells by RT-PCR. Site-directed mutagenesis using QuickChange II Mutagenesis Kit (Stratagene, Catalog, no. 200523) was performed after cloning into pBluescriptII(KS) (Stratagene, Catalog, no. 200455). cDNAs were fused to a 3×Flag-tag at their 3′ end. WT and mutated alleles of Cyclin A2 were verified by sequencing.

NIH3T3 (ATCC, No. CRL-1658), HEK 293 (ATCC, No. CRL-1573), MEFs, *p27^−/−^* MEFs (kind gift from K. Nakayama, Nippon Roche Research Center, Kanagawa, Japan), *p107^−/−^* MEFs (kind gift from Julien Sage, Stanford University Medical Center), *CDK2* ^−/−^, double *CDK2* ^−/−^ *p27* ^−/−^ and *p21* ^−/−^ 3T3 (kind gift from M. Barbacid, CNIO, Madrid, Spain) cells were maintained in DMEM medium with 10% fetal calf serum (Biowest). Transfections were performed using Lipofectamine 2000 (Invitrogen, Catalog no. 11668-019).

### Co-immunoprecipitation and kinase assay

Immunoprecipitations were performed using anti-Flag M2 affinity gel (Sigma, Catalog no. A2220) and precipitates were either resuspended in Laemmli buffer for western blot analysis or washed additionally with reaction buffer (20 mM Tris pH 7.5 and 7.5 mM MgCl_2_) for the kinase assay. Immunoprecipitated complexes were tested for kinase activity as previously described [23]. Experiments were repeated 5 and 3 times in NIH3T3 and HEK 293 cells, respectively. Binding affinities and kinase activity were quantified using ImageQuantTL software, normalized with respect to immunoprecipitated Cyclin A2 and represented as percentage relative to the WT allele taken as 100%.

### Antibodies, immunoblotting and immunoflorescence

The following antibodies were used for immunoblotting: monoclonal anti-Cyclin A2 (clone CY-A1 –Sigma, Catalog no. C4710), monoclonal anti-Flag antibody (Clone M2, Sigma, Catalog no. F9291), anti-CDK2 (Santa Cruz Biotechnology, Catalog no. sc-163), anti-CDK1 (mouse monoclonal, BD Biosciences, Catalog no. C12720-050), anti-p107 (Santa Cruz Biotechnology, Catalog no. sc-318.), anti-p21 (Santa Cruz Biotechnology, Catalog no. sc-397-G), anti-p27 (Santa Cruz Biotechnology, Catalog no. sc-528-G), Secondary HRP conjugated antibodies were obtained from Pierce. All immunoblottings were repeated at least 3 times.

The ant-Flag antibody (Clone M2, Sigma, Catalog no. F9291) and anti-Cyclin A2 (S. Cruz (H-432) Catalog no. sc-751) with corresponding AlexaFluor (Invitrogen) secondary antibodies were used for immunofluorescence staining. Prior to immunofluorescence analysis, cells were fixed with 3.2% paraformaldehyde and permeabilised with 0,2% triton X-100 (anti-Flag) or −20° acetone (anti-Cyclin A2). After 1 h blocking in 5% BSA cells were incubated 30 min with 1∶500 or 1∶1000 dilution of primary anti-Flag or anti-Cyclin A2 antibody respectively. Secondary antibodies were obtained from Invitrogen-Molecular Probes. Cells were visualized using Leica DMRA microscope and MetaMorph software. Three independent immunofluorescence analyses were performed.
